# Human Milk Microbiome and Microbiome-Related Products: Potential Modulators of Infant Growth

**DOI:** 10.3390/nu14235148

**Published:** 2022-12-03

**Authors:** Jie Ma, Debra J. Palmer, Donna Geddes, Ching Tat Lai, Lisa Stinson

**Affiliations:** 1 School of Molecular Sciences, University of Western Australia, Perth, WA 6009, Australia; 2 Telethon Kids Institute, University of Western Australia, Perth, WA 6009, Australia; 3 School of Medicine, University of Western Australia, Perth, WA 6009, Australia

**Keywords:** infant growth, human milk, human milk microbiome, infant gut microbiome, human milk oligosaccharides, short chain fatty acids, lactoferrin, lysozyme

## Abstract

Infant growth trajectory may influence later-life obesity. Human milk provides a wide range of nutritional and bioactive components that are vital for infant growth. Compared to formula-fed infants, breastfed infants are less likely to develop later-onset obesity, highlighting the potential role of bioactive components present in human milk. Components of particular interest are the human milk microbiota, human milk oligosaccharides (HMOs), short-chain fatty acids (SCFAs), and antimicrobial proteins, each of which influence the infant gut microbiome, which in turn has been associated with infant body composition. SCFAs and antimicrobial proteins from human milk may also systemically influence infant metabolism. Although inconsistent, multiple studies have reported associations between HMOs and infant growth, while studies on other bioactive components in relation to infant growth are sparse. Moreover, these microbiome-related components may interact with each other within the mammary gland. Here, we review the evidence around the impact of human milk microbes, HMOs, SCFAs, and antimicrobial proteins on infant growth. Breastfeeding is a unique window of opportunity to promote optimal infant growth, with aberrant growth trajectories potentially creating short- and long-term public health burdens. Therefore, it is important to understand how bioactive components of human milk influence infant growth.

## 1. Introduction

Infant growth trajectory impacts both short- and long-term health outcomes. Excessive weight gain during infancy has been associated with increased obesity risk, while stunted growth has also been associated with obesity, along with delayed cognitive and motor skill development and increased mortality [1,2,3,4,5]. Infant body mass index (BMI) trajectories as early as 1 year can predict childhood obesity at 5 years of age [6]. Half of obese children and 70–80% of obese adolescents remain obese into adulthood [7]; therefore, early higher infant BMI can be a risk factor for later-life obesity. In addition, infants who have catch-up growth during infancy are also more likely to develop obesity [5], suggesting that enduring biological changes may occur during disturbances of infant growth trajectories.

**Figure 1:**
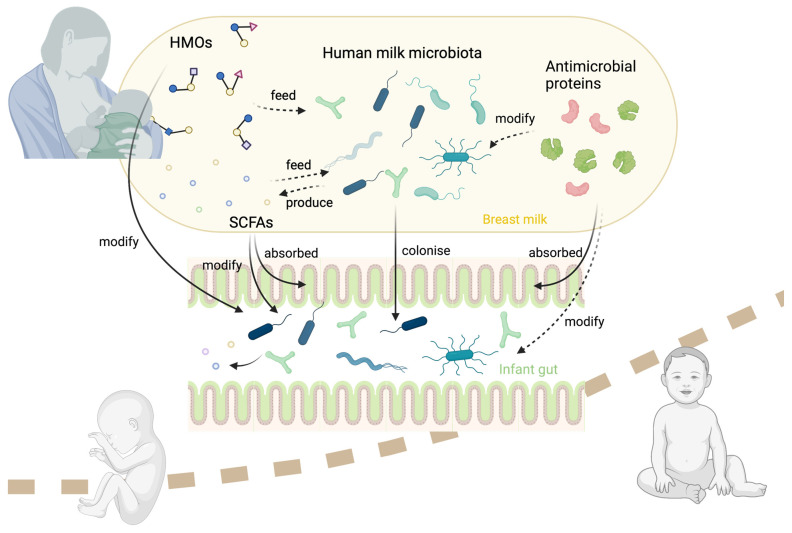
Potential interactions between human milk oligosaccharides (HMOs), microbiota, antimicrobial proteins, and short-chain fatty acids (SCFAs), within the mammary gland, and their influence on infant growth via the infant gut microbiota.

As the optimal source of nutrients for infants, human milk is recommended by the World Health Organisation as the exclusive food source for the first 6 months of life. In addition to its protective effect against a wide array of transmissible and noncommunicable diseases, breastfeeding also promotes healthy infant growth through its macro- and micronutrient content (including carbohydrates, fat, protein, fatty acids, vitamins, and minerals) and bioactive content (including hormones, growth factors, cytokines, microbes, metabolites, and oligosaccharides) [8,9,10]. Given increasing evidence demonstrating a link between the early-life microbiome and later-life body composition, this review will discuss the influence of the milk microbiota and microbiome-modulating factors (human milk oligosaccharides (HMOs), milk short-chain fatty acids (SCFAs), and antimicrobial proteins) on infant growth (Figure 1). In particular, we will focus on the potential of these components of human milk to modulate the infant gut microbiome, and the role of the gut microbiome in infant growth. By synergising data on various microbiome-related human milk components, this narrative review will provide novel insights into the impact of human milk on infant growth. Importantly, given that these microbiome and microbial-related components may interact within the lactating breast, their influence on the infant gut microbiome and growth should be considered holistically.

## 2. Review Methodology

A search was performed in PubMed (English) with the following keywords: human/breast milk microbiome, human/breast milk oligosaccharides, human/breast milk short-chain fatty acids, short-chain fatty acids, lactoferrin, lysozyme, breastfeeding, infant growth, infant body composition, infant anthropometrics, infant weight, and infant gut microbiome. The search covers the period prior to November 2022.

## 3. The Infant Gut Microbiome Can Influence Infant Growth

The gut microbiota has been demonstrated to affect body composition via mechanisms including energy harvesting and metabolic signalling [11,12]. Interestingly, animal studies have revealed that antibiotic treatment in infancy can alter the gut microbiota and lead to adiposity, and that adiposity persists to adulthood after the gut microbiota has recovered [13,14]. Similar results are also reported in human studies [15,16]. Therefore, it is likely that the early gut microbiota can influence infant growth and exert lifelong impacts on host metabolism.

Multiple large cohort studies have reported an association between the infant gut microbiota and growth [17,18,19,20,21]. Higher levels of *Bacteroides* spp., especially *Bacteroides fragilis*, and lower levels of Staphylococcus have been consistently reported to be associated with higher BMI in infancy, and to be predictive of childhood obesity [17,18,20,21]. One study of 49 children observed lower numbers of faecal Bifidobacterium and higher numbers of faecal *Staphylococcus aureus* at 6 and 12 months in infants who later developed overweight and obesity at 7 years of age [22]. In the CHILD cohort, increased diversity (richness) of the infant gut microbiota at 3–4 months of age was associated with higher risk of overweight at 1 year of age [23]. Conversely, another study of the same cohort later found that lower gut microbial diversity (Shannon index) at 1 year of age was associated with a rapid increase in BMI over the first 5 years of age [24]. The disparate results from these two studies may be due to the different time points at which faecal samples were analysed. Indeed, in the same cohort, diversity at 3 months was negatively associated with diversity at 1 year, perhaps suggesting that high diversity in early life may hamper the colonisation of new bacteria later in infancy [24]. While the relationship between the infant gut microbiome and growth remains complex, it may be that the cumulative change in the gut microbiome, rather than the state of the microbiome at a particular time point, influences infant growth [24]. Indeed, the primary influence may be the development of the gut microbiome as an ecological system and the sequential order of colonisation and succession within the infant gut. From this perspective, factors that influence the fitness of the microbiome, such as substrates for and products of bacterial metabolism and antibacterial compounds, should be considered when analysing the relationship between the infant gut microbiome and growth. An early window of opportunity exists for the development of the gut microbiota in relation to body composition, and perturbation during this period can lay the foundations for differing growth trajectories for life.

## 4. Development of Infant Gut Microbiota and Breastfeeding

Although the “sterile womb” theory is widely accepted, recent findings suggested that infants may be exposed to a low titre of microbes prior to delivery, with viable bacteria and bacterial DNA identified in placentas and amniotic fluid from healthy-term pregnancies [25,26]. However, this theory remains controversial, with opposing results also published [27,28]. Regardless of whether microbial exposure begins in utero, neonates harbour a low-biomass and low-diversity gut microbiome at birth, which develops in terms of diversity, complexity, and microbial load over time until a relatively stable stage is reached [29,30]. However, due to the short-term study design of many studies of the early-life gut microbiome (typically 1–3 years), it is difficult to say when the gut microbiome “matures” to an adult-like state. Recent evidence suggests that the gut microbiome of school-age children (5–12 years), differs significantly to that of adults in terms of both composition and function [31,32,33]. Gut microbiome development is therefore likely to be a gradual process that continues throughout childhood. Nonetheless, the first 2–3 years of life appear to be a particularly dynamic and unstable time within the gut microbiome [31,32,33,34]. From birth, the richness and complexity of the infant gut microbiota gradually increases and eventually reaches a stage that is relatively ecologically stable. During this time, the composition of infant gut microbiome can be influenced by multiple factors, including mode of delivery at birth, gestational age, feeding mode, maternal diet, and antibiotic usage [35,36,37,38,39]. Of all these factors, breastfeeding has been reported to be the most impactful factor, followed by birth mode [32]. Similarly, in another large cohort study of 903 children, intake of human milk was the primary factor associated with the composition and function of the infant gut microbiome [34]. Additionally, consumption of human milk was associated with lower diversity and higher levels of *Bifidobacterium* spp. in the infant gut [34]. While higher gut microbiome diversity is generally considered optimal for adults, low gut diversity is considered optimal during infancy, especially during breastfeeding. Infants who are breastfed exhibit lower gut microbiome diversity than those who receive formula [23,40], with a strong dominance of Bifidobacterium species [41,42,43,44]. Therefore, early maturation, predicted by higher diversity, may potentially impact the overall development of the gut microbiota negatively. Another study showed that the cessation of breastfeeding, rather than the introduction of solid food, is pivotal in infant gut microbiome diversification, highlighting the role of the bioactive components in human milk [45]. These bioactive components include HMOs, microbes, short-chain fatty acids, and antimicrobial proteins, which together influence the assembly of the infant gut microbiome and infant growth.

## 5. The Human Milk Microbiome

The human milk microbiome comprises all kingdoms of microorganisms, including bacteria, archaea, microeukaryotes, and viruses, with the bacterial community being the most abundant and well characterised [45]. These microbes may originate from maternal body sites (gut, skin, or the secretions from the pregnant mammary gland) [45,46,47] or from the infant oral cavity via retrograde flow during milk ejections that occur during breastfeeding [48]. In human milk, the most prevalent and abundant bacterial genera are Streptococcus and Staphylococcus, which together typically make up over half of the total profile [49]. Other taxa vary between populations and geographical locations, likely due to maternal and environmental factors, and only constitute a small portion of the milk microbiota [50].

### 5.1. Human Milk Microbes Colonise the Infant Gut

The infant gut microbiota are acquired both vertically from mother to infant, and horizontally from shared environments and social contacts [51]. One important function of human milk microbiota is the contribution via vertical transmission of microbes from mother to infant, as a small number of shared bacterial strains have been repetitively identified in mother–infant pairs, particularly bifidobacterial strains [52,53,54,55]. Milani et al. reported vertical transmission of *Bifidobacterium breve* and *Bifidobacterium longum* subsp. *longum* strains from milk to the infant gut, which was confirmed by strain isolation and whole-genome sequencing [55]. While *B*. *longum* was present in both maternal stool and milk samples, *B. breve* was only found in milk but not in the maternal gut, although it may have been undetectable in the maternal gut due to low abundance [55]. *B. breve* has been reported to only contribute to 0.07% of the maternal gut microbiota, but 28.44% and 67.7% of the milk and infant gut microbiota, respectively [56]. However, it should be noted that the mechanism by which these microbes are transferred from the maternal gut to the mammary gland remains unclear. Interestingly, these two species, *B. longum* and *B. breve*, together dominate the bifidobacterial genus of exclusively breastfed infants and persist in the infant gut at 6 months of age [57]. This implies a role of milk microbes in the development of the infant gut microbiome, although strain-level studies repeatedly identify only a very small number of shared taxa [46,52,55,58].

### 5.2. The Potential Role of the Human Milk Microbiota in Infant Growth

The human milk microbiome may influence infant growth by shaping the infant gut microbiota, as indicated by albeit weak evidence to date. Currently, only one study has assessed the relationship between the human milk microbiota and infant growth. Cheema et al. reported significant associations between the milk microbiome and infant body composition at 3 months of age (n = 60) [59]. In this study, the relative abundances of *Staphylococcus epidermidis*, *Streptococcus parasanguis*, and *Streptococcus lactarius* were positively associated with infant anthropometry, adiposity, and fat-free mass, and *S. epidermidis* was negatively associated with infant length. Associations between the milk microbiome and infant body composition also varied by maternal HMO secretor status. In infants of non-secretor mothers (those which lack the Se gene for the production of fucosylated HMOs), *Streptococcus mitis* was negatively associated with anthropometry and *S. parasanguis* was positively associated with BMI-for-age z-score. Interestingly, they also reported associations between maternal anthropometry and body composition with specific milk microbes. Maternal weight, fat mass, fat-free mass, and fat mass index were negatively associated with the relative abundance of *S. epidermidis*, and fat-free mass was positively associated with *Veillonella nakazawae*. These results indicate a possible cross-generation transmission of body composition via milk microbiota. Future research should include matching human milk and infant faecal samples with whole-genome sequencing analysis, along with human milk HMO composition, to increase our evidence on associations between the human milk microbiome and composition as a mediator of infant growth.

### 5.3. The Milk Microbiome as a Potential Contributor to the Intergenerational Transmission of Body Composition

Altered gut bacterial patterns observed in overweight mothers are echoed in their milk microbiome and in infants who later become overweight. Collado et al. reported that the total counts of Bacteroides and Staphylococcus were significantly higher in the gut of overweight women, as measured by fluorescent in situ hybridisation (FISH) and quantitative polymerase chain reaction (qPCR) [60]. During pregnancy, women who experienced higher weight gain had higher counts of Bacteroides and lower counts of Bifidobacterium in their gut. In the same overweight population, higher counts of Staphylococcus and Lactobacillus and lower counts of Bifidobacteria were found in milk samples collected at 1 month and 6 months postnatally [61]. Similarly, lower counts of Bifidobacteria and higher counts of *Staphylococcus aureus* findings were reported in the stool samples of a group of infants who later became overweight in childhood [22]. These findings suggest that overweight mothers harbor a distinct gut microbiota profile that is reflected in their milk microbiome. Such differences in the maternal microbiome may have consequences for infant gut microbiome colonisation and body composition. However, it remains unclear whether this similarity in the gut microbiome of overweight mothers and their infants who later become overweight is transmitted via the milk microbiome or influenced by other factors such as a shared environment.

Current knowledge implies a plausible route for the transmission of body composition from mother to offspring: overweight and obesity status may change the maternal gut microbiome during pregnancy, which may influence the infant gut microbiome via the milk microbiota during breastfeeding, and ultimately impact infant growth. However, while studies support the notion that gut bacteria influence infant growth [17,19], and that bacteria are vertically transferred from mother to infant via milk [55], the effects of horizontal transmission and host–gene interactions on the development of infant gut microbiota are also likely important [51].

## 6. Human Milk Oligosaccharides (HMOs)

Beyond the milk microbiome, other components in milk, such as HMOs, have additional effects on the infant gut microbiome and potentially infant growth. HMOs are a group of structurally distinct glycans in human milk [62]. They are the third most abundant solid in human milk after lactose and lipids, and are more abundant than protein [63]. More than 200 different HMOs have been identified, though a group of 19 make up more than 90% of the HMO profile [64]. The composition and concentration of HMOs varies between each mother and across lactation [65,66]. Genetically, the HMO profile can be classified into four groups according to the expression of the genes Se and Le, which are responsible for the expression of two enzymes involved in the synthesis of fucosylated HMOs, α1-2-fucosyltransferase (FUT2) (encoded by the Se gene) and α1-3/4-fucosyltransferase (FUT3) (encoded by the Le gene) [62]. Although the nongenetic factors that influence HMO composition remain largely unknown, some studies have suggested that HMO composition is associated with stage of lactation [67,68,69,70,71], maternal diet [43,70,72,73,74,75,76], and maternal BMI [59,70,73,77,78,79].

Despite the high abundance of HMOs in human milk, they are largely indigestible by the infant. Instead, they serve as an energy source for the microbiota in the infant gut, mainly Bifidobacterium, which degrade HMOs to create metabolites, including short-chain fatty acids (SCFAs) [64]. A recent in vitro study cultivated infant gut microbiota together with three groups of HMO(s), 2′FL only, 2′FL + LNnT, and a mixture of six HMOs (2′FL, LNnT, LNT, diFL, 3′SL, 6′SL) [80]. They found that although all HMOs increased SCFA levels, only the latter two promoted the growth of Bifidobacterium, while the predominant SCFA producer, Ruminococcus, was particularly boosted by the group of six HMOs. These results suggest that HMOs may function synergistically to have a greater influence on colonisation of the infant gut microbiome.

To degrade HMOs into monosaccharides, multiple enzymes from microbes are required for the breakdown of different linkages. In the gut, certain bacterial species express the whole set of enzymes for the digestion of all HMOs, such as *Bifidobacterium infantis, Bacteroides fragilis,* and *Bacteroides vulgatus*, while some are only capable of metabolising a subset of the HMOs, such as *Bifidobacterium breve* and *Bifidobacterium longum* [81]. The breakdown of HMOs usually occurs in an ordered manner, with modifications removed before the core structures can be degraded [82]. Therefore, some bacteria, such as *B. breve*, can only function in the presence of bacteria that perform the preceding breakdown steps [83]. This cross-feeding behaviour, especially in the bifidobacterial genus, results in a synergistic thriving of beneficial bacteria. During breastfeeding, these HMO-consuming bacteria thrive, and as a result, suppress the growth of other bacteria, including potential pathogens. Therefore, each mother’s characteristic HMO profile may shape the infant gut microbiome in an individualised way.

### HMOs Are Associated with Infant Growth

Multiple studies, although inconsistent, have identified various associations between individual HMOs and/or HMO diversity and infant growth [77,83,84,85,86,87,88,89,90,91]. Most of the previous studies have measured HMOs using high-performance liquid chromatography–mass spectrometry (HPLC-MS) in conjunction with fluorescent derivatisation [59,77,78,84,85,86,87,88,90,91], and two have used nano-LC-chip/time-of-flight (TOF)-MS [83,89]. However, differences in study populations and design, including sample time point(s) and reporting styles for infant growth data, make it difficult to identify any consensus between studies, with contradictory associations reported for some HMOs (Table 1).

Associations between infant anthropometrics and the most abundant HMO, 3′SL (3′ sialyllactose), are widely reported [59,77,78,83,86,87,89,90]. Concentrations of 3′SL have been positively associated with infant weight [89], length [77], head circumference [77,83], weight for length [88], fat mass [78], and fat-free mass [59,90], except in one study that reported a negative association with infant length [88]. This discrepancy may be due to the time at which infant anthropometrics were measured (birth to 4 months in one study, and 5 and 9 months in the other) [77,88]. Further research is needed to determine if the relationship between HMOs and infant body composition changes over the course of lactation.

Further, relationships are likely to vary depending on whether HMO concentration or intake is measured [45,59]. Intake of 3′SL at 2 months of age has been positively associated with infant weight, length, and fat-free mass, which is consistent with studies measuring 3′SL concentrations [59,78,90]. However, results in relation to intake are not always consistent with that of the concentration. While 3′SL intake was also associated with infant fat mass [78], such association has not been reported in concentration studies. Cheema et al. measured both HMO concentrations and intakes and found that except for 3′SL, none of the associations between HMO intakes and infant growth held true for concentrations, suggesting that concentration itself is not adequate for assessing associations between HMO and infant growth [59]. However, results also differ between studies in which intakes were measured. While both Saben et al. and Cheema et al. identified positive associations between 3′SL and 3FL (3 fucosyllactose) and infant growth, 6′SL (6′ sialyllactose) was positively associated with infant fat mass in one study and negatively in the other [59,78]. Similarly, many associations were identified in one study but not the other. This discrepancy is likely due to differences in the measurement of milk intake [59,78]. Saben et al. estimated the intake by measuring body weight before and after one feed and the feeding frequency, while Cheema et al. measured the total intake over 24 h. Nevertheless, the collective evidence demonstrates that infant intake of HMOs appears to be one of the factors that can regulate infant growth.

Maternal secretor status influences HMO composition, particularly 2′FL (2′ fucosyllactose) and LNnT (lacto-N-neotetraose), both of which have been added to commercial formulas [92]. 2′FL concentration has been positively associated with infant weight [77,87], length [89,90], and fat mass [77] in both healthy and malnourished populations. 2′FL intake also has been positively associated with weight and fat mass [59]. Another typical secretor, HMO LNnT, which is negatively correlated with 2′FL, is negatively associated with infant weight [77,87], length [86,87], BMI [77], fat mass [77], and fat mass percentage [85]. An opposing result with infant weight gain has also been reported, but only in non-secretor mothers [84]. Although some studies have reported different outcomes between individual HMOs and infant growth depending on maternal secretor status, secretor status by itself does not predict infant growth trajectory. Therefore, the role of HMOs in infant growth may be independent of secretion status, or potentially these results have been impaired by measuring concentrations instead of total infant intakes.

## 7. Short-Chain Fatty Acids

Short-chain fatty acids are the microbial metabolites of fibre fermentation that are produced in the colon. Some SCFAs, particularly butyrate, are absorbed locally by colonocytes, while the rest are transported to the portal vein and metabolised by the liver or distributed systemically around the body [93]. Although only a small concentration of SCFAs enter the peripheral circulation, they participate in a wide range of metabolic processes by regulating gene expression and binding to G-protein-coupled receptors (GPRs) [94]. In adults, SCFAs have been proposed to affect appetite control, energy harvesting, energy expenditure, and glucose homeostasis [95,96]. In vitro, acetate, butyrate, and propionate have been shown to stimulate the production of the satiety hormone peptide YY and glucagon-like peptide 1 [97,98]. Rodent studies have indicated that acetate can cross the blood–brain barrier and reach the hypothalamus, where it suppresses appetite [99]. Moreover, greater levels of energy expenditure after SCFA administration have been observed in both mouse studies and human studies [100,101,102] indicating potential for interventions designed to optimise the development of body composition early in life. This evidence supports the direct influence of SCFAs on host metabolism through multiple mechanisms.

In addition to direct influence, SCFAs may also indirectly influence host metabolism by impacting the gut microbiome. SCFAs are not only products of but also substrates for microbial metabolisms. Unlike acetate, which can be produced by a wide range of bacteria, pathways for propionate and butyrate production are relatively conserved in a few bacterial genera [103,104]. During the production of SCFAs, intermediate products such as succinate and lactate will be further utilised by a subset of bacteria. Additionally, acetate can be directly utilised by the butyrate-producing bacteria through the acetyl-CoA pathway [105]. This cross-feeding behaviour during the production of SCFA has been shown to promote the growth of certain bacteria and the diversity of microbiota in the gut, which in turn may influence host metabolism [106].

### Human Milk SCFAs and Infant Growth

The SCFAs acetate, butyrate, and formate have been identified in human milk [107,108,109]. Given that SCFAs participate in host metabolism, it is likely that SCFAs in human milk may influence infant growth. Currently, only one study has assessed the associations between milk SCFA levels and infant growth outcomes. Prentice et al. analysed SCFA levels in milk samples taken 4–8 weeks postpartum (N = 619) [107]. Child weight, length, and skinfold thicknesses (triceps, subscapular, flank, quadriceps) were measured at 3, 12, and 24 months of age. Human milk butyrate was negatively associated with infant BMI and skinfold thicknesses at 12 months of age, formate was negatively associated with infant BMI at all time points, and acetate was negatively associated with infant skinfold thickness at 3 and 24 months of age. These results highlighted the potential of SCFAs in human milk to influence infant growth, even beyond the period of exclusive breast feeding.

However, due to the dearth of studies on human milk SCFAs, their functions and mechanisms of action remain largely unclear. SCFAs may be transported to the milk from the maternal gut via the circulatory system, or they may be produced locally since milk contains both microbes and HMOs. Compared to the SCFAs identified in faeces and blood, the major SCFA propionate has not been identified in human milk [107,108,109]. Current studies focus on the function of SCFAs produced in the colon, but whether they can survive when consumed by infants in milk and reach the gut intact remains unknown. Therefore, more mechanistic studies are needed to identify both the source and function of human milk SCFAs.

## 8. Antimicrobial Proteins—Lactoferrin and Lysozyme

Lactoferrin and lysozyme are two of the most studied antimicrobial proteins in human milk [110]. Lactoferrin functions as carrier of iron in human milk [111,112]. It has been shown to inhibit infections of pathogenic bacteria [113,114,115], viruses [116,117,118], fungi [119,120,121], and parasites [122,123,124]. Conversely, lactoferrin peptides also have a strong bifidogenic effect and promote some species of lactobacilli, both of which are thought of as beneficial infant gut taxa [125,126,127]. As one of the major enzymes of human milk, lysozyme lyses Gram-positive bacteria, and when in corporation with lactoferrin, some Gram-negative bacteria as well [128,129]. A piglet model has shown that consumption of lysozyme-rich milk results in an increase in Bifidobacteriaceae and Lactobacillaceae and a decrease in pathogens in the gut [130]. The influence of lysozyme on the gut microbiome was also identified in a drosophila model [131]. Therefore, these antimicrobial proteins may influence infant growth indirectly through the modification of the infant gut microbiome. Apart from their antimicrobial properties, lactoferrin also stimulates intestinal and bone cell proliferation [132,133,134], which may directly influence infant growth.

Evidence for lactoferrin and lysozyme influencing infant growth is sparse. Bovine lactoferrin supplementation in formula (N = 10–12) has been associated with increased infant weight (6 months) and length (4 and 6 months) [135]. Similar results (increased length/height) were reported in a group of older children (12–36 months; N = 26) who received direct bovine lactoferrin supplementation [136]. However, it is difficult to make any conclusions as human milk lactoferrin is not comparable to bovine lactoferrin. Lysozyme supplementation of donor milk (N = 64) and higher concentrations in human milk (N = 42) are both associated with increased infant weight [137,138]. Gridneva et al. measured the total intake of lactoferrin and lysozyme (N = 20; 2–12 months) and found that the intake of lactoferrin was negatively associated with fat-free mass and lysozyme positively with fat mass [139]. Although weak evidence supports the associations between lactoferrin and lysozyme and infant growth, the mechanisms remain to be unveiled. Further studies are needed to elucidate the association between antimicrobial proteins in human milk and infant growth.

## 9. Interactions of Microbiome and Microbiome-Related Components within the Lactating Mammary Gland

As both HMOs and SCFAs can influence the infant gut microbiota, they may also modify the human milk microbiota through the same mechanisms. HMOs may feed a certain group of bacteria in human milk, creating SCFAs as a by-product (Figure 1). SCFAs may also be metabolised by bacteria within the lactating mammary gland. In addition, as known for their antimicrobial property, lactoferrin and lysozyme may also participate in this interaction.

Although it is currently unclear whether digestion of HMOs occurs within the mammary gland, several studies have suggested that the composition of HMOs is associated with the milk microbiota composition. Aakko et al. observed a positive correlation between total HMO concentration and counts of *Bifidobacterium* spp. in colostrum in a small study (N = 11) [140]. This association was more pronounced when HMOs were grouped by structure. Positive associations were identified between *B. breve* and sialylated HMOs and between *B. longum* and non-fucosylated/non-sialylated HMOs. Similarly, another larger cohort study has demonstrated an association between milk microbiota and individual HMOs (N = 393) [141]. Specifically, the relative abundance of Bifidobacterium was negatively associated with the concentration of DLNH, and in non-secretors only the relative abundance of Staphylococcus was positively associated with concentration of sialylated HMOs. These findings together imply a potential role of HMO in modifying the milk microbiota before reaching the infant gut.

Unlike studies in HMOs, to date no study has examined the associations between microbiota and SCFAs in human milk. If SCFAs are produced from bacterial HMO fermentation within the mammary gland, they may be associated with both the HMO and microbiota content of human milk. Milk SCFAs may also act as a substrate for milk bacterial metabolism independently of HMOs.

Although currently no studies have been carried out to assess the influence of lactoferrin and lysozyme on the human milk microbiome, they also have the potential to influence microbiome composition for their antimicrobial properties. They may interact with the human milk microbiota in a similar manner as in the gut microbiome, as shown in animal and human studies [130,131]. Like other microbiome-related products in human milk, antimicrobial proteins are likely to participate in the regulation of microbiota in human milk and infant gut.

Further studies are needed to elucidate the association between milk SCFAs, microbiota, HMOs, and antimicrobial proteins. If they interact with each other within the mammary gland, they may influence infant growth in an integrated manner. This highlights the importance of viewing lactation as a biological system, with human milk as a whole, promoting healthy infant growth. This may also partially explain why associations between HMOs and infant growth/body composition are inconsistent, as other human milk components may be involved.

## 10. Summary and Future Directions

In this review, we summarised the current evidence regarding the potential function of the human milk microbiome and microbiome-related products on infant growth, potentially via the modification of the infant gut microbiome and other mechanisms. As these components may interact with one another, they may synergistically influence the human milk microbiome, the infant gut microbiome, and infant growth. While numerous studies have assessed the influence of HMOs on infant growth, evidence for other components is limited. Given the fact that the infant gut microbiome is dynamic in early life, longitudinal studies of larger sample sizes are needed to assess associations between these milk components, the infant microbiome, and infant growth. Importantly, given the potential interactions between these components, an integrative approach is required to holistically assess the impact of human milk on infant growth. This review also highlighted the importance of analysing intakes rather than concentrations of human milk components. Future studies should measure daily intake when possible, to more accurately assess the impact of human milk components on infant growth. Integrated longitudinal studies are required to ascertain the influence of the human milk microbiota and microbiome-related products on growth throughout infancy and beyond, with a focus on their interactions with the infant gut microbiome.

## Figures and Tables

**Table 1:** Summary of associations between HMOs and infant growth outcomes. Weight includes growth outcomes of weight z score (weight compared to a standard of the same sex), change in weight z score, weight for age z score (weight compared to a standard of the same age and sex), and change in weight for age z score; length includes length z score, change in length z score, length for age z score, and change in length for age z score. Weight for length includes both weight for length z score (weight compared to a standard of the same length and sex) and change in weight for length z score. Head circumference includes head circumference z score, change in head circumference z score, head circumference for age z score, and change in head circumference for age z score. Body mass index includes growth outcomes of body mass index and body mass index for age z score. Fat mass includes fat mass, percentage of fat mass (weight of body fat), and fat mass index (calculated as (fat mass)/height^2^). Fat-free mass includes fat-free mass (weight of body par that do not contain fat), percentage of fat-free mass, and fat-free mass index (calculated as (fat-free mass)/height^2^).

Anthropometrics	
Weight	**Positive** 3′SL * [59,78,87,89], LNnT [84], 2′FL * [59,77,87], LNFP II [78], 3FL * [59,78,87], DSLNT [84], DFLac * [59,77,87], LSTb ** [78], DFLNH * [59], DSLNH ** [78], DFLNT [59], 6′GL [91]
	**Negative** LNnT [77,87], 6′SL ** [59], LNFP II [84], LNT [83], LNFP I [85], LSTb [87], LSTc [89], DFLNH [77], MFLNH III [91]
Length	**Positive** 3′SL * [59,86], LNnT [86], 2′FL [87,90], 3FL ** [59], DFLNH * [59], A-tetra [90], DFLNT [59], LNnDFH [91]
	**Negative** 3′SL [88], LNnT [59,86,87,88], LNT [86], 3FL [91], LNFP I [86], LSTb [87], MFLNH III [91], LNFP V [86], FLNH [86]
Weight for length	**Positive** 3′SL [88], LNFP II [78], LNT [78], 3FL ** [78], LSTb ** [78], LSTc [88], LDFT [83], IFLNH 1 [83]
	**Negative** LNFP II [83], LNT [83], LSTa [83], DFLNHc [83]
Head circumference	**Positive** 3′SL [77,83], 6′SL [90], LNFP III [88], DFLac [77], MFLNH III [88], LDFT [91], A-tetra [88], LNDFH I [91], LNnDFH [91]
	**Negative** 6′SL [83], LNFP III [89], LNT [83], LNFP I [89], DFLNH [77], MFLNH III [91], LNnFP [86], DFLNHa [89], LNFP V [86]
Body mass index	**Positive** DFLac ** [59], LSTb ** [59], DFLNT ** [59]
	**Negative** LNnT [87], 2′FL [86], 6′SL [77], LNT [86], LNnFP [86], LNFP V [86]
Body composition	
Fat mass	**Positive** 3′SL ** [78], 2′FL * [59], 6′SL ** [78], LNFP III ** [78], LNFP II * [78,85], DSLNT * [78,85], LSTb ** [78], FDSLNH [85], DSLNH ** [78], DFLNT [59]
	**Negative** LNnT [77,85], 6′SL ** [59], LNFP III ** [59] LNFP I [85], DFLNH [77]
Fat-free mass	**Positive** 3′SL * [59,90], 3FL ** [59], DFLac ** [59], DFLNH * [59], DFLNT ** [59]
	**Negative** LNT [90], LNFP I [85], LSTc [90]
Fat mass/fat-free mass ratio	**Negative** LNFP III ** [59]

Abbreviations: 3′SL: 3′-Sialyllactose, LNnT: Lacto-n-neotatraose, 2’FL: 2′-Fucosyllactose, 6’SL: 6′-Sialyllactose, LNFP III: Lacto-N-fucopentaose III, LNFP II: Lacto-N-fucopentaose II, LNT: Lacto-N-Tetraose, 3FL: 3-Fucosyllactose, DSLNT: disialyllacto-N-tetraose, DFLac: difucosyllactose, LNFP I: Lacto-N-fucopentaose I, LSTb: sialyl-lacto-N-tetraose, LSTc: sialyl-lacto-N-tetraose, DFLNH: Difucosyllacto-N-hexaose, MFLNH III: Monofucosyllacto-N-hexaose III, LDFT: lactodifucotetraose, A-tetra: A-tetrasaccharide, IFLNH I: fucosyl-paralacto-N-hexaose I, FDSLNH: Fucosyl-disialyllacto-N-hexose, DSLNH: disialyllacto-N-hexaose, DFLNT: difucosyllacto-N-tetrose, LNDFH I: Lacto-N-difucohexaose I, LNnDFH: lactoN-neodifucohexaose, 6′GL: 6′galactosyllactose, LNnFP: lacto-N-neofucopentaose, DFLNHa: difucosyllacto-N-hexaose (a), LSTa: LS-Tetrasaccharide a, DFLNHc: difucosyllacto-N-hexaose c, LNFP V: lacto-N-fucopentaose-V, FLNH: fucosyllacto-N-hexaose. * Associations with both concentration and infant intake. ** Associations with infant intake only.

## Data Availability

Not applicable.
